# Supplementary material for: Cerebrospinal Fluid Neurofilaments Light-Chain Differentiate Patients Affected by Alzheimer’s Disease with Different Rate of Progression (RoP): A Preliminary Study
Source: Brain Sci. 2024 Sep 25;14(10):960. doi: 10.3390/brainsci14100960 (PMC11505946; doi:10.3390/brainsci14100960)
Supplement: Supplementary file 1 [file brainsci-14-00960-s001.zip › brainsci-3214385-supplementary.pdf]

Supplemental Materials

Article

Cerebrospinal fluid Neurofilaments light-chain differentiate patients affected by Alzheimer's disease with different rate of progression (RoP): a preliminary study

Table S-1. Demographic and clinical features of all participants. Continuous variables are expressed as median with interquartile ranges (IQR), while categorical variables are expressed as relative frequencies.

**Variables**	**AD (n=56)**
Age at onset (*years*)	69 (64 – 72)
Age at LP[A](*years*)	73 (68 – 77)
Gender (% *male*)	55.3
Education (*years*)	8 (5 – 13)
Diagnostic delay (*years*)	4 (3 – 6)
Follow up (*years*)	1.25 (1.00 – 2.00)
MMSE[B] (*raw scores*)	22 (17 – 25)
History of dementia (%)	66.1
Apo E ε4 (%)	42.8
RoP (*scores*)	2.0 (-0.6 – 3.8)
Memory onset (%)	83.9

[A] Lumbar Puncture; [B] Mini Mental Status Examination.

Table S-2. Relationship between RoP and demographic or clinical features of participants investigated by Spearman's correlation analyses.

**RoP** *vs*	**rho**	***p***
Age at onset	0.058	0.670
Age at LP[A]	-0.006	0.967
Education	0.130	0.338
Diagnostic delay	0.165	0.255
Follow up	0.137	0.314
MMSE[B]	-0.045	0.740

[A] Lumbar puncture; [B] Mini Mental State examination.

Table S-3. Logistic regression analysis to investigate the predictive roles of demographic and clinical features of participants in contributing to scores of RoP upper than median value (RoP U-M: >2). Bold font indicates a statistical significance (p<0.05).

**RoP U-M (cost.)**	**B**	**s.e.**[1]	**DF**[2]	**OR (95%C.I.)**	***p***
Age at onset	-0.012	0.047	1	0.988 (0.002 – 1.082)	0.793
Age at LP	0.011	0.047	1	1.011 (0.922 – 1.110)	0.812
Gender (F)	0.250	0.553	1	1.284 (0.434 – 3.717)	0.651
Education	0.020	0.061	1	1.020 (0.905 – 1.150)	0.741
Diagnostic delay	0.160	0.119	1	1.173 (0.928 – 1.483)	0.181
MMSE	-0.027	0.049	1	0.974 (0.284 – 1.072)	0.587
Follow-up	0.542	0.404	1	1.720 (0.779 – 3.797)	0.180
Apo E ε4 (yes)	-0.131	0.554	1	0.887 (0.296 – 2.599)	0.813
History of dementia (yes)	-0.613	0.584	1	0.542 (0.173 – 1.701)	0.294
Memory onset (yes)	-0.305	0.767	1	0.737 (0.164 – 3.313)	0.691
Therapy (yes)	-0.033	0.957	1	0.938 (0.148 – 6.313)	0.973
Sleep disturbances (yes)	-0.791	0.736	1	0.453 (0.107 – 1.919)	0.283
Hypertension (yes)	-0.250	0.533	1	0.779 (0.263 – 2.303)	0.651
Heart diseases (yes)	-0.560	0.657	1	0.571 (0.158 – 2.071)	0.394
Diabetes mellitus (yes)	0.305	0.767	1	1.357 (0.302 – 6.103)	0.691
Dyslipidemia (yes)	-0.113	0.593	1	0.893 (0.279 – 2.852)	0.848
Cerebrovascular diseases (yes)	0.316	0.581	1	1.317 (0.439 – 4.287)	0.587
Psychotic disorders (yes)	0.088	0.788	1	1.092 (0.233 – 5.118)	0.911

[1] s.e. = standard error; [2] DF= degree of freedom.
